# Visual narratives in medicine – Bridging the gap in graphic medicine with an illustrated narrative of osteoarthritis

**DOI:** 10.1016/j.ocarto.2024.100471

**Published:** 2024-04-18

**Authors:** Vicky Duong, Samantha Bunzli, Leigh F. Callahan, Corné Baatenburg de Jong, David J. Hunter, Jason S. Kim, Ali Mobasheri

**Affiliations:** a Sydney Musculoskeletal Health, The Kolling Institute, Northern Clinical School, The University of Sydney, Sydney, NSW, Australia; b School of Health Sciences and Social Work, Griffith University, Nathan Campus, Queensland, Australia; c Physiotherapy Department, Royal Brisbane and Women's Hospital, Queensland, Australia; d Thurston Research Center, Osteoarthritis Action Alliance, University of North Carolina, North Carolina, USA; e ReumaNederland, Amsterdam, the Netherlands; f Arthritis Foundation, USA; g Research Unit of Health Sciences and Technology, Faculty of Medicine, University of Oulu, Oulu, Finland; h Department of Regenerative Medicine, State Research Institute Centre for Innovative Medicine, Vilnius, Lithuania; i Department of Joint Surgery, First Affiliated Hospital of Sun Yat-sen University, Guangzhou, China; j World Health Organization Collaborating Centre for Public Health Aspects of Musculoskeletal Health and Aging, Université de Liège, Liège, Belgium

**Keywords:** Osteoarthritis, Visual narratives, Discourse, Comics, Narrative

## Abstract

**Objective:**

Visual narratives have been used in medicine to share information in the form of stories with the potential to improve understanding of conditions and change behaviours. One genre of visual narratives is “graphic medicine”, which integrates comics into medical education and the delivery of healthcare. Graphic medicine can maximise the impact of research findings by presenting them in a more accessible format, which may be particularly useful in certain populations, such as those with low levels of health literacy. Those with lower health literacy levels and osteoarthritis (OA) are less likely to manage their condition with guideline recommended management strategies, experience a higher burden of disease, and have lower access to care. Our objectives were to review the current visual narratives in the field of and create a graphic medicine visual narrative based on existing research.

**Design:**

This paper summarises the current visual narratives in OA and presents a graphic medicine visual narrative to illustrate the experience of living with OA. Considerations for the dissemination of visual narratives to target audiences are also discussed.

**Results:**

The most common visual narratives in are infographics, videos, and graphic medicine. A graphic medicine visual narrative, based on previous qualitative work and informed by a framework, was created to illustrate two distinct narratives – impairment and participatory.

**Conclusion:**

Visual narratives remain an emerging field in OA but may serve as a useful resource for patients or clinicians to discuss various aspects of OA management. Future research should evaluate and validate the use of visual narratives in OA.

## 1 Introduction

Osteoarthritis (OA) is the most common form of joint disease and a leading cause of disability worldwide [1]. It is estimated that approximately 10–12% of the adult population have symptomatic OA [2,3]. Osteoarthritis most commonly affects the load-bearing joints such as the hip and knees, as well as the hands. In the absence of a cure for OA, current management strategies are focussed on alleviating symptoms using core treatments of self-management, exercise, physical activity, weight management or weight loss if overweight/obese. These core treatments are advocated by OA guidelines and appropriate for all individuals with OA, regardless of the stage of OA, or severity of symptoms [[4], [5], [6]].

Despite the extensive research and international guidelines advocating for these core treatments, the uptake amongst people with OA is low [7]. This is driven in part, by poor access to evidence-based information due to a combination of factors, including costs associated with accessing care for OA; long waiting lists; inadequate training of health professionals; low-quality and/or conflicting information about OA online. These factors particularly impact those most affected by the social determinants of health, such as people with lower levels of health literacy. People with lower levels of health literacy are less likely to manage their OA with guideline recommended management strategies, suffer a higher burden of disease and have lower access to care [7].

Education resources can be provided as a stand-alone, or as part of a consultation to empower people with OA and their families to participate in shared decision making about care, engage in healthy behaviour change and active self-management to positively impact OA outcomes [8]. Resources that are tailored to meet the needs of people with OA, are likely to lead to greater uptake of the recommendations within them [8,9]. While substantial efforts are underway to tailor the content of education resources in a way that can address knowledge gaps and misconceptions about OA, less attention has been paid to the format of education resources and in particular, how these may be tailored to meet the needs and preferences of people with lower levels of health literacy.

Narrative communication is defined as “a representation of connected events and characters which have an identifiable structure, is bounded in space and time, and contains implicit or explicit messages about the topic addressed” [10]. This contrasts with a didactic style of communication which merely presents information in the form of reasons and evidence to support a claim. In the context of OA, there has been a shift from paternalistic and didactic approaches of communication towards a shared, 2-way partnership between the clinician and patient to empower patients about their care. Visual narratives are the combination of narrative communication and a visual component. Visual narratives are a method for communicating health information to a range of audiences with varying levels of health literacy. Visual narratives are usually sequential images, which convey a continuous event sequence, and typically tell a story [11]. A common type of visual narrative in the field of medicine and health are patient narratives, defined as “illustrative accounts of individual patients’ experiences [with a certain illness]” [12]. However, visual narratives can also provide perspectives from caregivers or health care practitioners. Shaffer has synthesised the five main purposes of narratives in healthcare and their respective outcomes which include providing information about various health care concerns, and using an alternative format which is easier to comprehend [12]. Cognitive learning theories suggest that narratives may be more effective for communicating information about health care, including radiographs, treatment efficacy, anatomy as the information is processed more effectively compared to didactic information [13]. Other purposes include creating more engaging and relevant health materials, which has been shown to be particularly useful in populations of low health literacy [14,15], model target behaviours, persuade healthy behaviours and providing comfort to patients and families [12]. This format of communicating health information has been shown to improve evidence-based knowledge and uptake of evidence-based recommendations in serious pathologies such as cancer [[16], [17], [18]], as well as public health pandemics such as COVID-19 and AIDS [19,20].

There are many formats of visual narratives; public visual narratives can be advertisements, public health campaigns, whereas personal visual narratives could be patient stories in the form of videos or other graphic formats. Visual narratives can be used to communicate to a variety of audiences including patients and patient groups, medical students, health care practitioners, researchers, policy makers and government and regulatory agencies. The aim of this paper is to review current visual narratives in the field of OA and explore how existing research can be harnessed to create more compelling visual narratives for patient populations. Considerations for the development and dissemination of visual narratives to this target audience will also be discussed.

## 2 Visual narratives in medicine and osteoarthritis

There are three main types of visual narratives: infographics, videos, and graphic medicine. These are discussed in turn below and summarised in Table 1.

**Table 1:** Summary of visual narratives in osteoarthritis.

Modality and description	Main aim	Primary intended audience	Advantages	Disadvantages	Examples in osteoarthritis
Infographic	Research dissemination	Researchers Health care providers Policy makers	Ability to summarise large amounts of data and share widely Allows research findings to be communicated in a more engaging way compared to traditional outputs Usually evidence-based	Often do not present a patient narrative which limits engagement	“Improved care for osteoarthritis: the forgotten chronic disease” by The Osteoarthritis Research Society International [35] British Journal of Sports Medicine: “Running with OA” [36] “Changing the narrative around OA care” [37]
Videos	Patient education	Consumers	Provide information, engage, model behaviour, persuade and provide comfort [23] Provide support by sharing similar experiences [24] Serve as a resource when preparing for important health care decisions [25]	Treatment effects are difficult to evaluate due to the lack of standardisation in patient narratives [23]	The Arthritis Foundation (USA) MyJointPain website (www.myjointpain.org.au), an OA self-management website founded in Australia. Staying Strong with Arthritis – personal accounts from Aboriginal and Torres Strait Islander people living with arthritis [38]
Graphic medicine	Patient education	Consumers Health care providers	Improved comprehension, enjoyable to consume [20] Ability to translate to various languages due to limited text	Graphical structure (e.g. sequential images) not be understood by all ages or cultures [11]	None that we are aware of

### 2.1 Infographics

An infographic, short for ‘information graphic’ is a “visualisation of data and ideas which conveys complex information to an audience in a manner that can be quickly consumed and easily understood” [21]. Infographics are increasingly used in the field of research and policy and act as a potential solution to improve the barriers of traditional academic outputs, such as difficulties with access, as well as comprehension, lack of engagement and time required to consume text-heavy formats [22].

### 2.2 Videos

Patient narratives in the form of videos are illustrative accounts of individual patients' experiences with a certain illness [12]. The main purposes of web-based videos include: providing information, engaging, modelling behaviour, persuading, and providing comfort [23]. Videos can provide support by sharing similar experiences [24] or serve as a resource when preparing for important health care decisions [25]. These narratives aim to build a community through online support groups and connections with others living with arthritis. However, due to a lack of standardisation in patient narratives, their treatment effects are difficult to evaluate [23]. Guidelines from the database of patients’ experiences (DIPEx) have been created in an attempt to improve the rigour of qualitative research about patient experiences [26].

### 2.3 Graphic medicine

Graphic medicine uses visual storytelling in the form of comic art to share health-related information and experiences [27]. Graphic medicine has been used to illustrate the patient journey of living with certain conditions – from the initial diagnosis to trialling treatments, but also provides clinicians and health care providers greater insights into the patient experience [28]. Graphic medicine has been used for decades, particularly in the field of public health to communicate information ranging from skin cancer [18] to HIV/AIDS [19]. Graphic medicine is also heavily present in the field of cancer, with examples such as Cancer Vixen [17] and Mom's Cancer [16], recounting people's true experience with the disease. The use of graphic medicine has been used by public health professionals to convey messages to younger audiences and those with low literacy [19,29]. The influence of graphic medicine has been understudied, with the majority of research focussing on the process of creation and interpretative meaning rather than its effect [30]. Although there is a lack of research into the best forms of dissemination, a recent case-crossover study evaluated study participant preferences and found preliminary evidence that comics were preferred over a traditional lay summary and scientific abstract control [20]. Reasons for this preference included improved comprehension and that they were enjoyable to consume [20]. A randomized pilot trial comparing the use of graphic medicine to improve patient comprehension and periprocedural anxiety before coronary interventions found that a consent form including graphic illustrations improved comprehension (higher scores in all three subcategories of comprehension items: procedural details, risks and behavioural measures after the procedure) as well as decreased periprocedural anxiety across time points [31].

Despite the assumed comprehension of sequential images in a comic format, this format is not recognised by all. Preliminary work by Cohn suggests that the comprehension of visual narratives requires a fluency which develops over age and exposure [11]. In certain cultures, sequential images may be interpreted as a single, isolated scene for example, in Nepal [32], Papua New Guinea [33] and Aboriginal Australian Umpila speakers [34].

Visual narratives remain an emerging field in OA and a unique opportunity exists for new work in this field to improve the patient narrative of OA. In the next section, we present suggestions for developing a graphic medicine narrative of OA.

## 3 Developing a graphic medicine narrative of osteoarthritis

Most narratives in OA research have focussed on verbal narratives, highlighting how people communicate about the condition. A synthesis of qualitative research involving people experiencing OA, their carers, and clinicians, identified two overarching ways of talking about OA: 1) an “impairment narrative” – that frames pain and disability associated with osteoarthritis as a consequence of a broken ‘body machine’ which has a ‘use-by date’ and can only be ‘repaired’ by a doctor; and 2) a “participatory narrative” – that focuses on what people can do to remain active despite signs and symptoms of OA and empowers people with OA as experts in their own lived experience and agents for change [39]. These findings provide insights into how people with OA think about their condition and why they act as they do. For example, Bunzli et al. found that use of an impairment narrative may influence acceptance and adoption of evidence-based OA treatments among patients and clinicians by perpetuating misconceptions such as “exercise can damage a joint with signs and symptoms of OA” [40]. These findings provide clinicians and researchers with an opportunity to reframe how they communicate to patients with OA to encourage better management of the condition, prioritising core treatments such as self-management, education, exercise, physical activity, and weight management/loss.

Below we translate these findings into a graphic medicine format. Adopting a framework proposed by Meuschke [41] we first identified ‘story pieces’ that represent the content of the story (i.e. impairment and participatory narratives); ‘narrative characters’ (i.e. a patient, seeking care from two different clinicians and their friend); a ‘conflict’ describing the challenges the character(s) encounter on their health journey (unhelpful information from the clinician and found online); and a ‘structure’ from introduction to climatic peak to resolution, in which the patient is provided with a helpful narrative to make sense of their OA.

**Fig. 1:**
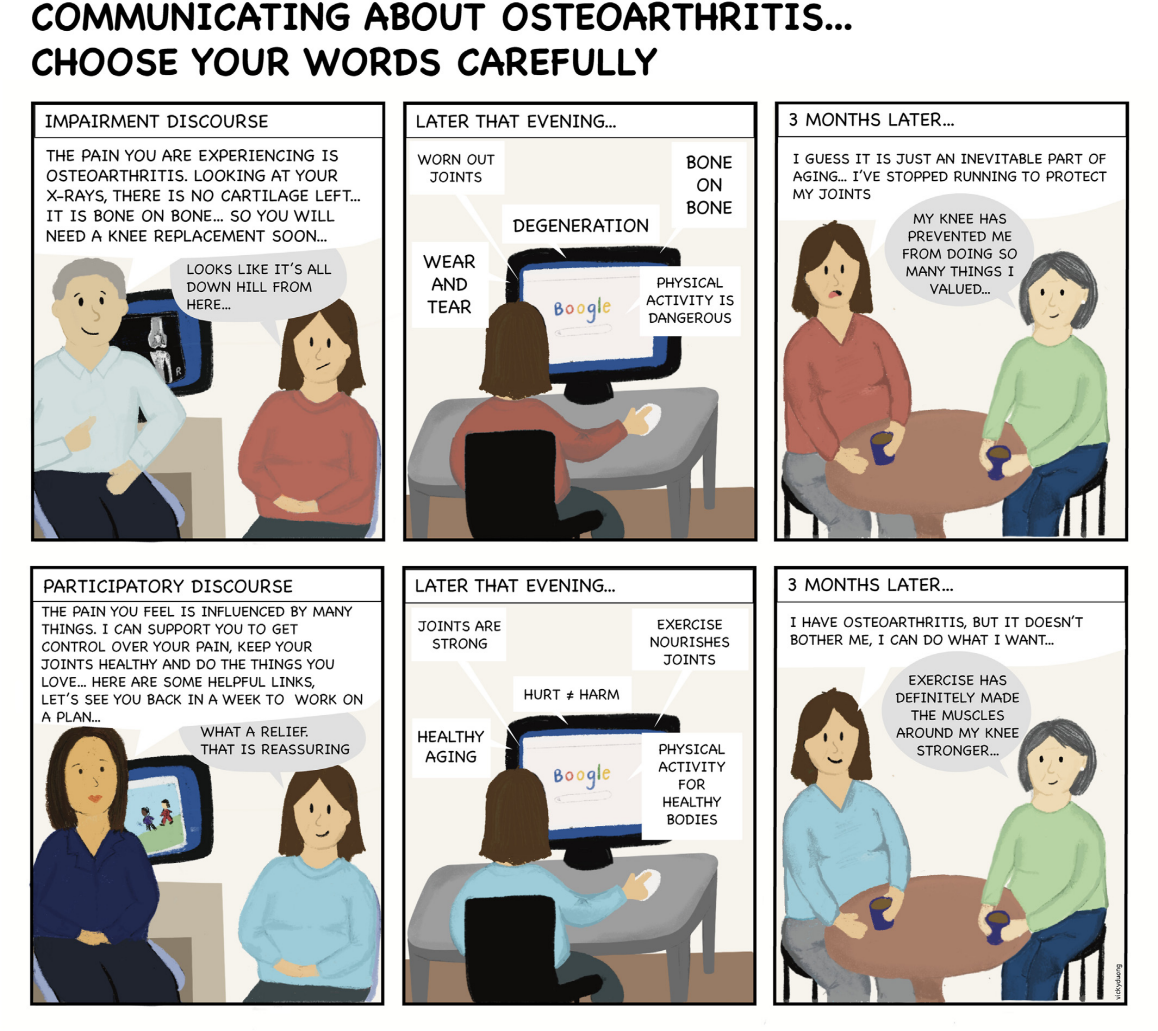
A graphic medicine example of two different narratives for osteoarthritis.

By illustrating an impairment and participatory narrative side by side, the aim of the comic is to demonstrate how different language can be perceived by people seeking care. Although the information presented in graphic medicine can be similar to information presented textually, we believe that the combination of text and the visual component conveys the narrative of OA diagnosis in a simple and comprehensible manner. We focussed on a single comic which would not only highlight the patient experience but also serve as a communication medium for clinicians to explain certain aspects of OA management, shedding insight on the feelings of overwhelming anxiety and confusion that may also arise with a diagnosis of OA. The final output was a series of three images documenting the initial diagnosis of OA, highlighting two different narratives (Fig. 1). The purpose of this comic was for illustrative purposes only; however, with future consumer input, this comic may be adapted for diverse audiences, including people with different levels of health literacy, and translated into other languages. Further evaluation of the comic in improving understanding and beliefs of OA would support the use of visual narratives in future OA research and in clinical practice.

## 4 Considerations for creating visual narratives in OA

Guidelines for the creation of infographics with public health messaging have been previously created by Public Health England [42], many of which are applicable to visual narratives the context of OA patients and patient groups. A few key features are discussed below:

1. Target your audience - The language and the use of data visualisation presented should be targeted to your audience. Ideally, visual narratives should be co-designed with consumers, with consumer input at all stages, from design to dissemination.
2. Devise a key message - Consider whether you want to present an overview of a topic or focus on a specific aspect of the patient-journey to encourage behaviour change, provide comfort, or change beliefs.
3. Representation - It is important that your narrative depicts your target audience. Some considerations include the age of OA diagnosis, lifestyle factors, cultural and linguistic diversity, setting (urban/rural).
4. Evaluation and revise – Seek feedback from your target audience to ensure that your visual narrative is appropriate, appealing, understandable, and serves it's intended purpose. Visual misinformation, whereby information can be presented in a way that is misleading or out of context is of growing concern [30], and co-design from the target audience can help to improve this.
5. Dissemination *-* When disseminating your visual narrative, consider appropriate channels for your target audience. Accessibility to resources should also be considered. It is noted that individuals with lower health literacy are less likely to seek health-related information on the internet [43] and this may further exacerbate disparities in health and healthcare. The development of digital skills may enable individuals to use digital technology more effectively.

Although visual narratives in the field of OA are still emerging, the majority of the existing resources have not been robustly evaluated or created with consumer and end-user input. There is a need to evaluate the effectiveness of visual narratives in order to best communicate OA education to people living with OA, their carers, as well as to clinicians and researchers. This will ensure that research and up-to-date knowledge about best-evidence OA management is more effectively translated into clinical practice.

## 5 Conclusion

Visual narratives have been present in the field of medicine and are increasingly used to convey information to various audiences. The most common forms of visual narratives in OA are infographics and videos. There is great potential for the use of visual narratives, particularly graphic medicine in the field of OA, where patient beliefs about the condition negatively impact the uptake of evidence-based treatments. A graphic medicine example highlighting OA beliefs was created based on existing evidence-based research and may serve as a resource for patients or clinicians to share knowledge, discuss appropriate treatment expectations and reconsider the language they use when communicating about OA. Visual narratives paint compelling stories about the patients’ lived experiences with a condition. The creation of visual narratives should be thoughtful and be targeted to the intended audience, using appropriate language and context. Future research should identify and evaluate the optimal types of visual narratives for various OA audiences at different points along the patient journey.

## Funding

DJH is supported by an 10.13039/501100000925NHMRC Investigator Grant (APP1194737).

## Author contributions

VD and SB wrote the first draft of the manuscript. All authors contributed to the manuscript, revision, read and approved and final version.

## Conflict of interest

DJH has received consulting fees from Pfizer, Merck Serono, Kolon TissueGene, and TLC (<$10,000 each). AM has received consulting fees from Aptissen SA, GSK, GSK Consumer Healthcare/HALEON, Kolon TissueGene, Laboratoires Expanscience, Novartis, Orion Corporation, Viatris, Genacol, Sterifarma, Sanofi (Brazil), Janssen-Cilag (Brazil), Sanofi (France), Sanofi (USA), Pacira Biosciences, Grünenthal, Viatris, Novartis, Expanscience, Galapagos, Chiron, Dorian Therapeutics, Ampio Pharmaceuticals, Contura, Nestlé, Nestlé Wobenzym®, Synartro AB, Pfizer Consumer Healthcare and Aché Laboratórios Farmacêuticos (<$10,000 each). No other disclosures relevant to this article are declared.
